# Plasma *α*-Glutathione *S*-Transferase Evaluation in Patients with Acute and Chronic Liver Injury

**DOI:** 10.1155/2019/5850787

**Published:** 2019-10-20

**Authors:** Jolanta Czuczejko, Celestyna Mila-Kierzenkowska, Karolina Szewczyk-Golec

**Affiliations:** ^1^Department of Psychiatry, Ludwik Rydygier Collegium Medicum of Nicolaus Copernicus University, 9 M. Curie Skłodowskiej St, 85-094 Bydgoszcz, Poland; ^2^Department of Medical Biology and Biochemistry, Ludwik Rydygier Collegium Medicum of Nicolaus Copernicus University, 24 Karłowicza St, 85-092 Bydgoszcz, Poland

## Abstract

High concentration of alpha-glutathione *S*-transferase (*α*-GST) in the liver and its short half-life make this enzyme clinically useful in assessing hepatocellular damage. We aimed to investigate the significance of *α*-GST evaluation in monitoring of liver injury in acute and chronic liver diseases. 20 healthy volunteers and 52 patients were included in the study. The patients were divided into three groups: group I (acute viral hepatitis B or C), group II (chronic hepatitis B or C), and group III (chronic liver disease or cirrhosis with different etiologies). The concentration of *α*-GST and the activities of alanine aminotransferase (ALT) and aspartate aminotransferase (AST) were measured in all examined groups. *α*-GST, ALT, and AST were statistically higher in all patient groups than in the control group. Statistically higher values of all assessed parameters were observed in group I compared with remaining patients. Statistically higher activities of ALT and AST are observed in group III compared with group II. Significant positive correlations were noted between *α*-GST and ALT/AST in groups I and III. The results indicate that the assay of *α*-GST in combination with the other conventional markers may be found as a confirmatory test for hepatocellular damage.

## 1. Introduction

Glutathione *S*-transferases (GSTs; EC 2.5.1.18) are enzymes that generally catalyse the formation of conjugates between glutathione (GSH) and a wide variety of electrophilic compounds (carcinogens, toxins, and drugs) [1–6]. GSTs also demonstrate selenium-independent glutathione peroxidase activity and can catalyse reduction of organic hydroperoxides using GSH as a donor of -SH groups [2, 4]. The distinctive feature of GSTs is their occurrence as tissue-specific isoenzymes. GST isoenzymes have been identified in the liver, spleen, pancreas, lungs, heart, testicles, kidney, nervous tissue, erythrocytes, and placenta. In mammals, all cytosolic GSTs are dimeric. Based on amino-acid sequence similarities, there are seven classes of cytosolic GSTs: Alpha, Mu, Pi, Sigma, Theta, Omega, and Zeta (1), which exhibit different physical, chemical, immune, structural, and kinetic properties [6]. The Alpha and Mu classes can form heterodimers [1].

Alpha GSTs (*α*-GSTs and GSTA1) are ubiquitously present in all cell types, but at a particularly high concentration in hepatocytes (7). The main functions of *α*-GSTs in the liver are as follows: binding steroids, bile acids, and bilirubin, reduction of lipid peroxides, synthesis of prostaglandins and leukotrienes, transport of amino acids, and conjugation of electrophilic compounds (7). Alcoholism, as well as HBV and HCV infections, stimulates the immune system, production of free radicals, and detoxifying mechanisms and, in turn, may induce *α*-GST production in hepatocytes [7].

*α*-GSTs can be used as a more sensitive biomarker of liver function than the conventionally analysed aspartate aminotransferase (AST) and alanine aminotransferase (ALT) because of its lower molecular weight and shorter half-life (3). Multiple studies have pointed to *α*-GST as an earlier and better marker of liver injury in acetaminophen and alcohol poisoning, HBV (hepatitis B virus) and HCV (hepatitis C virus) infection, allograft rejection, and liver ischemic-reperfusion injury [2, 3, 5, 8–11]. The main disadvantage of aminotransferases is their uneven distribution in the liver [10, 12]. The periportal concentration of these enzymes is relatively higher than that in the centrilobular region, with the latter region being the most injury sensitive [12]. The centrilobular region is vulnerable to viral attack and is involved in the detoxification of drugs, xenobiotics, and alcohol [2, 10, 12, 13]. As opposed to aminotransferases, subunits of *α*-GST are evenly and abundantly distributed throughout the liver [2, 8, 10]. As much as 80% of the total *α*-GST content in the body is located in the liver. In a single hepatocyte, *α*-GST constitutes 3–5% of all soluble cytosolic proteins, whereas ALT is only 0.6% [2, 10, 12]. Considering the low molecular weight of *α*-GST (52 kDa) and its high liver concentration, great amounts of *α*-GST are very quickly released from injured hepatocytes into blood plasma [2, 3, 12, 13]. The half-life of *α*-GST is less than 60 minutes. Additionally, after *α*-GST is released to the plasma, its concentration returns to normal values after 5 days. These features of *α*-GST are very helpful in the detection and monitoring of liver injury [13]. As opposed to *α*-GST, ALT and AST have higher molecular mass (95 and 92 kDa, respectively), and after liver injury, these are released into plasma later than *α*-GST [2, 12, 14]. ALT is released from hepatocytes within 5 days and returns to normal values after 10 days, and the half-life of ALT is longer than that of *α*-GST (48–72 hours) [2]. In addition to hepatocytes, AST is released from damaged myocytes; thus, the ratio of serum AST to ALT can be used to differentiate liver damage from other organ damage [15].

There is some evidence that *α*-GST better correlates with histological changes than ALT [10] and that ALT level is not always correlated with liver damage and disease progression in patients with HCV infection [16]. Some researchers have also indicated that the activity of ALT very often does not change during liver injury, even when the injury is histologically proven [2]. According to Ozer et al. [15], there is a need to use more specific biomarkers of liver injury in conjunction with ALT and AST, which may be useful in pharmaceutical industry.

Because *α*-GST has been demonstrated to be an earlier and a more sensitive marker of hepatocyte injury than aminotransferases [1, 17, 18], the aim of our study was to establish whether the monitoring of *α*-GST in the blood plasma of patients with acute and chronic liver diseases could provide significant information that may be clinically useful together with or independently of the ALT and AST levels.

## 2. Materials and Methods

### 2.1. Participants

Fifty-two patients with liver injury treated at the Department of Infectious Diseases, Medical University in Bydgoszcz, Poland, were included in the study. The patients were divided into three groups according to the acuteness and etiology of liver injury: group I including 21 patients (aged 20–77 years, mean age 48 years) with acute viral hepatitis B or C, group II including 13 patients (aged 23–79 years, mean age 43 years) with chronic hepatitis B or C, and group III including 18 patients (aged 28–79 years, mean age 54 years) with chronic liver disease or cirrhosis with different etiologies (alcoholism and acetaminophen overdose). Control group was composed of 20 healthy volunteers (aged 46–54 years, mean age 48 years). All participants were evaluated using standard physical examination and routine clinical laboratory tests. Liver injury in the patients was confirmed by histological and laboratory examinations. All study participants provided their written consent. The study was approved by the Local Ethics Committee at the Medical University in Bydgoszcz, Poland.

### 2.2. Study Design and Biochemical Analysis

In the course of the study, all blood samples were collected from the median cubital vein into heparin-containing tubes (9 mL) to obtain plasma. Blood was taken from the patients on admission to the hospital before they received treatment. The samples were centrifuged (at 6,000*g* for 10 min at 4°C), and the separated plasma was assayed for the ALT and AST activities in a clinical laboratory using a commercially available kit produced by Roche. Normal ranges for plasma ALT and AST were 0–40 U/L and 0–37 U/L, respectively.

The concentration of alpha-glutathione *S*-transferase (*α*-GST) in plasma was determined using a quantitative enzyme immunoassay (Biotrin HEPKIT™-Alpha, Biotrin, Sinsheim-Reihen, Germany). 100 *μ*l of standards (0–40 *μ*g *α*-GST/L), positive controls, and plasma samples diluted 1 : 5 were added to microassay wells coated with anti-*α*-GST IgG. After 60 min of incubation with uniform shaking, the wells were washed and incubated for 30 min with anti-*α*-GST IgG conjugated to horseradish peroxidase, washed again, and incubated for 15 min with a substrate (tetramethylbenzidine solution). After stopping the reaction, the plate was immediately read at 450 nm, using 630 nm as a reference wavelength (ELISA plate reader). The resultant color intensity was proportional to the amount of *α*-GST present in the sample.

### 2.3. Statistical Analysis

All results were expressed as mean ± standard deviation. One-way analysis of variance followed by Tukey's post hoc test was performed to determine the significance of the obtained differences. A value of *p* < 0.005 was considered statistically significant. The Pearson correlations were used to find any relationship between the measured parameters.

## 3. Results

The activities of ALT and AST, and the concentration of *α*-GST were higher in a statistically significant manner in all examined groups of patients than in the control group (Table 1).

Significantly higher values of all examined parameters were observed in the group of patients with acute viral hepatitis B or C (group I) than in patients with chronic hepatitis B or C (group II) and those with chronic liver disease or cirrhosis with different etiologies (group III).

In a comparison of groups II and III, statistically higher activities of ALT and AST were observed in group III.

**Figure 1:**
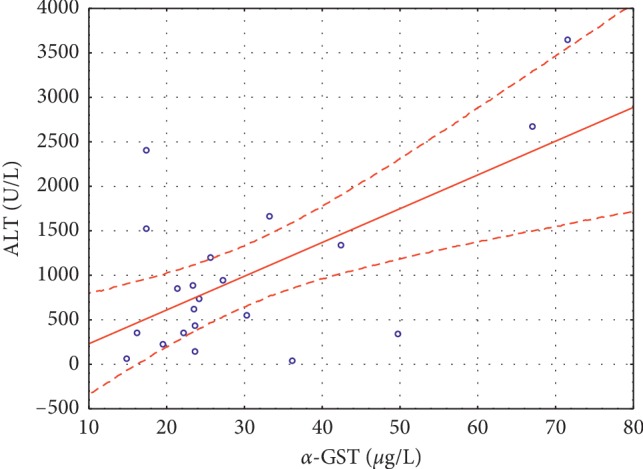
Significant positive correlations between *α*-GST and ALT (*r* = 0.3284 *p* < 0.002) in group I.

**Figure 2:**
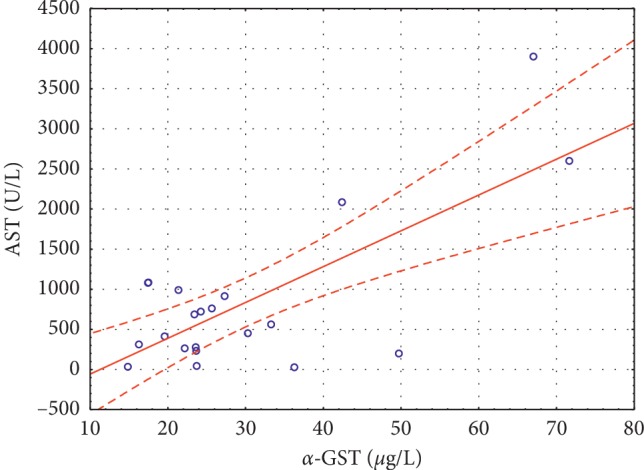
Significant positive correlations between *α*-GST and AST (*r* = 0.7311 *p* < 0.0001) in group I.

**Figure 3:**
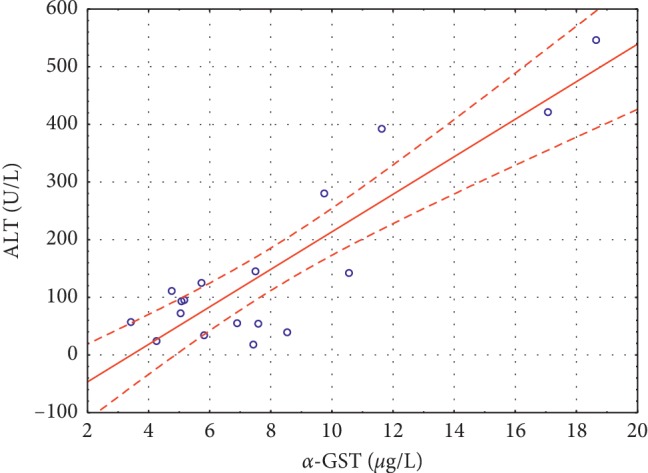
Significant positive correlations between *α*-GST and ALT (*r* = 0.8865 *p* < 0.0001) in group III.

**Figure 4:**
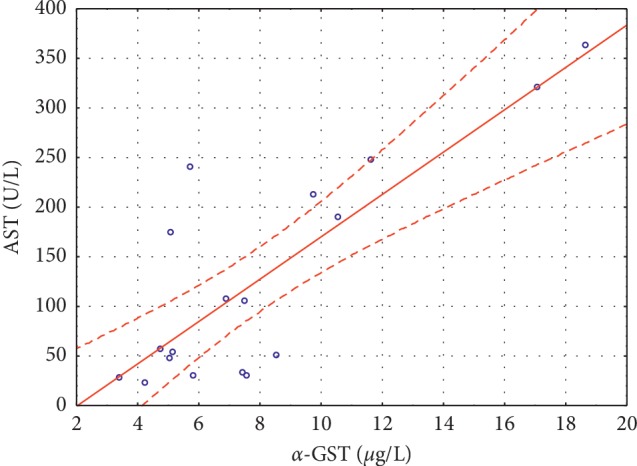
Significant positive correlations between *α*-GST and AST (*r* = 0.8175 *p* < 0.0001) in group III.

Significant positive correlations were noted between *α*-GST and ALT (*r* = 0.3284, *p* < 0.002) (Figure 1) and between *α*-GST and AST (*r* = 0.7311, *p* < 0.0001) (Figure 2) in group I, and between *α*-GST and ALT (*r* = 0.8865, *p* < 0.0001) (Figure 3), and *α*-GST and AST (*r* = 0.8175, *p* < 0.0001) (Figure 4) in group III. There were no correlations between the assessed enzymes in group II.

Table 2 presents multiplication factors by which the assessed enzymes increased in the patient groups compared to the control group. The highest increases in the ALT and AST activities and the concentration of *α*-GST were observed in group I. In the remaining groups, the activities of the assessed enzymes were higher in group III than in group II.

## 4. Discussion

The data obtained in our study indicate that *α*-GST can be a good marker of liver injury. However, the results of the *α*-GST assessment only supported the results of the aminotransferase assessment, as a significant increase in the *α*-GST concentration was observed only when the ALT and AST levels were increased. Both the concentration of *α*-GST and the ALT and AST activities were higher in a statistically significant manner compared to the control group, but the highest increase in the *α*-GST concentration observed in patients with acute viral hepatitis (6.8-fold) was still lower than that in ALT (48-fold) and AST (39.4-fold).

Abdel-Moneima and Sliemb [19] found a significant increase in the blood level of *α*-GST in all HCV groups compared with the control group. Even if normal ALT levels were observed, *α*-GST was significantly increased compared with the control [19]. Furthermore, the authors [19] noticed that the sensitivity of *α*-GST was similar to the sensitivity of ALT in all HCV groups and higher than the sensitivity of AST. Sídlová et al. [18] found significantly higher *α*-GST levels in patients with cystic fibrosis, even in those with normal results of standard liver tests and ultrasound scan, suggesting that serum *α*-GST is a more sensitive marker than transaminases for the detection of liver damage associated with cystic fibrosis. Similarly, Helaly and Mahmoud [20] found that *α*-GST concentrations were significantly above the normal range in 91.2% of HCV-infected participants compared with 4.4% for ALT and 17.6% for AST. In turn, Maina et al. [21] analysed ALT and AST activities and *α*-GST concentration in serum samples from patients with acetaminophen toxicity, drug-induced liver injury, ischemic hepatopathy, and autoimmune hepatitis. The authors obtained that median *α*-GST for all etiologies were increased on day 1, returning to normal by day 3, whereas median AST and ALT values did not return to normal, even at day 7. Based on these findings, they concluded that *α*-GST is a more sensitive marker of liver injury/recovery, allowing for more rapid real-time assessment of improvement or worsening of liver disease.

We found a significant positive correlation between *α*-GST and ALT and AST in the examined group of patients with acute viral hepatitis B or C, and this observation is similar to the results obtained by Abdel-Moneima and Sliemb [19]. The authors [19] also noticed an elevation of plasma *α*-GST in HCV patients with high ALT levels, but the sensitivity of *α*-GST in this group was lower than that of ALT and AST. They [19] claimed that these results could diminish the usefulness of assessment of *α*-GST compared with that of aminotransferases in patients with high ALT and that the measurement of *α*-GST would be more valuable in early detection of liver damage in HCV patients with normal ALT.

We also found a significant increase in *α*-GST, ALT, and AST in patients with chronic hepatitis B or C, and the elevation of the assessed enzymes was comparable to that in patients with acute hepatitis. However, the increase in *α*-GST, ALT, and AST was not as high as the increase in the assessed enzymes in the acute hepatitis group. According to Balistrei and Rej [22], a low increase and a normal value of *α*-GST, ALT, and AST could be explained by established liver cirrhosis, reduction of enzyme synthesis in hepatocytes, or malnutrition. Based on this hypothesis, in our study, ongoing hepatocyte injury was low in the examined patients with chronic hepatitis B or C.

In the plasma of patients with alcoholic cirrhosis, the activity of *α*-GST is often increased, even when transaminases are normal [7]. Beckett et al. [23] showed that 16 out of 19 patients with paracetamol poisoning had abnormal plasma GST concentrations, whereas the ALT activity was increased only in 8 patients. Additionally, an increase in the plasma GST concentration of more than 100 times the normal value was found in all severely poisoned patients, while the ALT activity was not increased (23). The authors [23] suggested that after a paracetamol overdose, there are two distinct phases of hepatotoxicity: early phase characterized by a small GST increase (within 4 hours) and a later progress marked by a 100-fold increase (up to 40 hours), and a second phase of hepatotoxicity with plasma GST concentrations increasing to 1000 times the upper limit of the reference range. Our data showed only two-fold increase in the plasma GST concentrations in the group of patients with paracetamol and alcohol overdose compared with the control group. Similarly, the increase in ALT (6.4-fold) and AST (5.7-fold) indicates an early phase of hepatotoxicity rather than severe liver damage. The obtained data were in agreement with the physician's diagnosis of chronic state of disease. We also noted significant positive correlations between *α*-GST and ALT, and *α*-GST and AST in the group of patients with chronic liver injury. We suggest that in our patients, the damage to hepatocytes was not as severe as in patients with acute disease, and therefore we did not find such spectacular increase in *α*-GST and aminotransferases. We also showed that in chronic patients, the increase in *α*-GST only accompanied the increase in ALT and AST, and thus *α*-GST did not prove to be a better marker of liver injury in those patients. Similarly, Federico et al. [7] stated that *α*-GST assessment adds further information with respect to other routine evaluations. However, they also noticed an increase in plasma *α*-GST in alcohol abusers and HCV-RNA positive patients with normal ALT levels and suggested that *α*-GST is more sensitive than ALT in detecting low-grade liver damage, especially in the case of centrilobular necrosis. Our results did not support this hypothesis. Federico et al. [7], following Venkatraman et al. [24], proposed that the lack of elevated *α*-GST in some alcoholics could be associated with a genetic pattern in those participants. Based on this hypothesis, we suggest that in our patients, there was a diminished expression of *α*-GST mRNA in the liver due to genetic conditioning.

According to our studies, measurement of *α*-GST could be considered as a useful confirmatory test for hepatocellular damage. However, high cost and complexity of the enzyme immunoassay used for the *α*-GST measurement in plasma make it difficult to use it in the routine diagnostic laboratory. Unfortunately, the cheaper and faster methods for *α*-GST seem to be not available. Habdous et al. [25] described very interesting, cheap and rapid spectrophotometric assay for serum total GST activity. Surprisingly, the levels of the enzyme calculated using this method were much higher than that found using radio- and enzyme immunoassays. Further analysis performed by Fabrini et al. [26] revealed that spectral changes interpreted as a measure of basal GST activity, were mainly due to the spontaneous reaction between the two substrates used. It was concluded that GST activity in normal serum cannot be correctly determined with the spectrophotometric assay mentioned because of the low enzyme concentration in serum and the pH-dependent artifacts.

## 5. Conclusion

The positive correlation found between *α*-GST and ALT and AST indicates that assessing *α*-GST in combination with conventional markers could be considered as a confirmatory test for hepatocellular damage. However, its role would be much more valuable in early detection of liver damage in patients with normal ALT. Taking into account high cost and complexity of *α*-GST assay when compared to cheap and rapid spectrophotometric methods for ALT and AST, the obtained data do not support the use of plasma *α*-GST as a better marker of liver damage than ALT and AST.

## Figures and Tables

**Table 1:** The activities of alanine aminotransferase (ALT) and aspartate aminotransferase (AST) and the concentration of alpha-glutathione *S-*transferase (*α*-GST) in the control group (healthy volunteers) compared with all examined groups.

	Control group	Patient groups		
		Group I	Group II	Group III
N	20	21	15	16
Age (years)	48 ± 3	52 ± 19	40 ± 15	55 ± 16
ALT (U/L)	25 ± 7	1201 ± 892^a^	66 ± 33^a,e^	159 ± 162^b,d,g^
AST (U/L)	23 ± 6	907 ± 913^b^	49 ± 37^c,d^	131 ± 115^b,f,g^
*α*-GST (*μ*g/L)	4.13 ± 1.9	27.99 ± 16.9^a^	11.28 ± 8.5^b,e^	8.42 ± 4.33^b,d^

*Note.* Group I: acute viral hepatitis B or C patients; group II: chronic hepatitis B or C patients; group III: patients with chronic liver disease or cirrhosis with different etiologies. All results are presented as a mean ± standard deviation. ^a^*p* < 0.0001, ^b^*p* < 0.001, ^c^*p* < 0.01 (control group vs. patient groups), ^d^*p* < 0.0001, ^e^*p* < 0.001, ^f^*p* < 0.01 (group I vs. group II or III), ^g^*p* < 0.05 (group II vs. group III).

**Table 2:** Increase in *α*-GST, ALT, and AST compared with the control group in all examined groups.

	Group I	Group II	Group III
ALT	48 ×	2.6 ×	6.4 ×
AST	39.4 ×	2.1 ×	5.7 ×
*α*-GST	6.8 ×	2.7 ×	2.0 ×

## Data Availability

The data that support the findings of this study are available on request from the corresponding author. The data are not publicly available because they contain information that could compromise research participant privacy/consent.
